# Stakeholder opinions on scientific forest management policy implementation in Nepal

**DOI:** 10.1371/journal.pone.0203106

**Published:** 2018-09-05

**Authors:** Omkar Joshi, Rajan Parajuli, Gehendra Kharel, Neelam C. Poudyal, Eric Taylor

**Affiliations:** 1 Department of Natural Resource Ecology and Management, Oklahoma State University, Stillwater, Oklahoma, United States of America; 2 Department of Forestry and Environmental Resources, North Carolina State University, Raleigh, North Carolina, United States of America; 3 Department of Forestry, Wildlife and Fisheries, University of Tennessee, Knoxville, Tennessee, United States of America; 4 Department of Sustainable Forestry, Texas A&M Forest Service, College Station, Texas, United States of America; University of Georgia, UNITED STATES

## Abstract

Despite its widespread recognition as a successful model of participatory forest management, the community forestry program in Nepal is often criticized for its protection-oriented emphasis. Recognizing the need for more active timber management, the government of Nepal recently adopted a scientific forest management (SFM) policy in the lowland tropical region. In this study, strength, weakness, opportunity, and threat analytical hierarchical process criteria were employed to understand stakeholder perceptions concerning SFM implementation in Nepal. The overall perception was prioritized in the order of strengths (35%), threats (28%), opportunities (22%), and weaknesses (16%). The study results suggest that there is agreement among stakeholders regarding the need for active management of forests in the tropical lowland region. However, the perceptions of academic researchers and non-government organization professionals differed from those of the other stakeholders in that those two groups were more concerned about potential corruption and uncertainties surrounding policy and legal issues. The findings suggest that the long-term success of SFM may depend on the ability of the government to develop a mechanism that is transparent and capable of ensuring equitable benefit sharing among stakeholders. While the stakeholder perception analysis performed in this study was focused on SFM implementation in Nepal, the results could have implications for other countries that practice the participatory model of forest governance as well.

## Introduction

**Fig 1:**
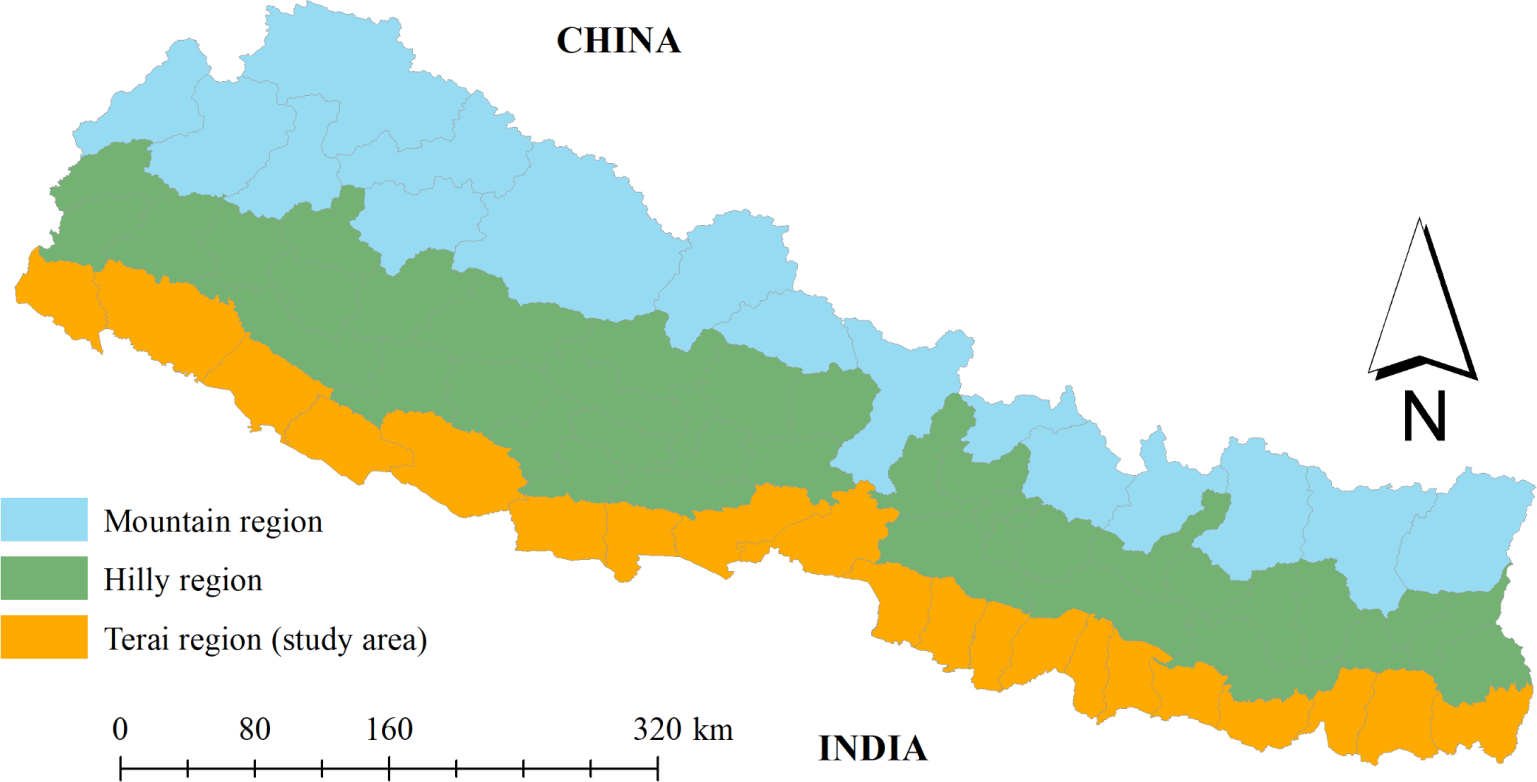
Geographic map of Nepal showing study area (Terai region).

Forests are one of the most accessible and reliable sources of revenue in the mid-hills and lowland tropical regions―commonly called Terai―in Nepal (Fig 1). The forest conditions in the mid-hills, once denuded due to massive deforestation, have gradually improved since the forest management system was transferred from government control to local control in the form of community forestry (CF) (e.g., [1–3]). The CF program in Nepal was institutionalized more than four decades ago by a legislative mechanism that allows the government to hand over usufruct rights of a part of public forests to local user groups, called community forest user groups (CFUGs) [1, 3, 4]. These user groups manage forests by following the guidelines outlined in a pre-approved forest operation plan and sharing benefits through the use and sale of forest products [3, 5]. Consequently, CF has become a vehicle of economic development in many rural communities in Nepal [1, 3].

While the CF program is internationally applauded for its strong focus on local participation and collective action, its success in Nepal is limited to the mid-hill region for several reasons. First, despite the continuous interest of the people of Terai, the government of Nepal has been reluctant to launch a CF program in the lowland region of Terai, which contains productive timberland with high commercial value (e.g., [2, 6–8]). Second, existing timber inventory guidelines for CF management, originally designed for the forest species of the mid-hills, have protection-oriented management priorities [9] and therefore cannot be implemented in the productive timberlands in lowland Terai without major modifications. Third, proper identification of true forest users is critical in forming dedicated CFUGs with a high level of social cohesiveness and trust [6, 10]. Unlike the mid-hills, the settlements in the lowland Terai regions are mostly occupied by a recent influx of migrants from the mid-hills, and these settlements are generally ethnically diverse and geographically distinct [6, 7, 10, 11]. In particular, dependency and use history are major criteria used to identify users, which is difficult to do among an immigrant population [10, 12]. Finally, stakeholders are also concerned that the commercially important Terai forests might further exacerbate governmental corruption and abuse of authority [13]. Consequently, only small patches of isolated forests are managed under the CF program in Terai [6].

For the aforementioned reasons, the government of Nepal has adopted different policies regarding Terai forest management. For example, during early 1990s, the government developed and launched an operational forest management plan in select Terai districts that focused on intensive timber harvesting and reforestation [7, 14]. Unfortunately, this production-oriented policy failed to receive public support, due partly to the fear that it would result in the felling of young or immature stands [7, 14]. Another kind of participatory forestry program, the collaborative forest (CBF) management program, was implemented in the past decade. Unlike CF, the CBF program was primarily designed to ensure that the traditional Madhesi population, who did not reside in the forests, received equal benefits from the Terai forest resources [7]. Of note, lowland Terai is also known as Madhes, as such the traditional population in this region, except the immigrants from mid-hills, are called the Madhesis [7]. Since migrant population from mid-hill resided near forests, traditional Madhesis were deprived of forest rights under traditional CF regime [7]. Unfortunately, the CBF program has also not received broad stakeholder support [6, 7, 13]. In particular, it was argued that some provisions of the CBF program undermined the autonomy of local users in forest management [6, 8].

### Multifaceted nature of participatory forestry programs in Nepal

While effective management of common pool forest resources has always been a challenge, there are several examples where communities have identified ways to govern common property regimes [15]. For example, southeast Asian countries such as Nepal, Vietnam, Cambodia, Indonesia, the Philippines, and Thailand have adopted participatory forest management models over the past few decades [16] and strive to integrate innovative scientific practices to increase productivity.

While the decentralization of power and authority has played an important role in the co-management of common pool resources [17], the failure of collective action introduces challenges into participatory management programs [18]. This is true in the cases of CF and CBF management in Nepal since the government, non-government organizations (NGOs), and forestry user groups, the primary stakeholders in the forestry sector of Nepal, have conflicting interests [7, 8, 18]. The government of Nepal, particularly the Department of Forests and its district units, is primarily involved with management and regulatory oversight [8]. Forestry user groups constitute another key stakeholder in forest management and benefit-sharing [8]. NGOs are largely concerned with advocacy and provide technical assistance to forest users. Similarly, academic and research communities are obvious contributors in typical policy processes, as they provide unbiased, science-based management information to the Nepalese forestry sector. Other stakeholders such as the media and private institutions also have roles in this sector [8].

Failure to manage the productive timberland of Terai scientifically is often considered to be a lost economic opportunity for Nepal. In addition, the gap between the timber demand and supply in Nepal has led to substantial timber importation and revenue loss [13, 14, 19], especially since the timber demand dramatically increased for building and reconstruction purposes after the devastating earthquake of 2015 [20]. As [19] revealed, more than 80% of the total timber demand in Nepal has been fulfilled by imports in the past two years.

In recent years, stakeholders have acknowledged that protection-oriented forest management cannot bring needed economic prosperity to a developing country like Nepal [13]. Therefore, the government recently initiated scientific forest management (SFM), which permits advanced silvicultural practices in the existing CFs and CBFs in eight of the Terai districts [14].

### SFM initiatives

SFM follows the typical forest management planning process, which includes (i) identification of the potential forest area; (ii) stakeholder interaction regarding the operational modality and benefit-sharing mechanism; (iii) forest boundary surveying and division of the area into blocks, compartments, and sub-compartments; (iv) assessment of forest cover, growing stock, and annual increment through inventory; and (v) stakeholder consultation and finalization of the management plan [14]. Silvicultural operations such as regeneration felling, thinning, and improvement felling within sub-compartments generate periodic revenue [14]. Equal numbers of trees from sub-compartments are harvested following yield regulations [14]. Based on these principles, approximately 26,000 hectares of Terai forests, both under CF and CBF programs, are currently being scientifically managed using SFM principles. Given the promising initial results, the government plans to extend SFM initiatives to other Terai districts as well [14].

Although SFM is a technically sound practice of active Terai forest management [14], scholars have argued that its sustainability and success will depend largely on positive collaboration and trust among key stakeholders [21]. In this study, we identified important issues related to SFM implementation in Nepal. We used the strengths, weaknesses, opportunities, and threats analytic hierarchical process (SWOT-AHP) framework to understand how stakeholders perceive the internal strengths and weaknesses as well as external opportunities and threats related to SFM practices in Nepal. While SWOT-AHP has been previously used to study Nepal’s community forestry program in general [22], our study, to the best of our knowledge, is the first effort to use this framework to understand stakeholder perceptions concerning recently introduced SFM implementation policy.

## Methods

### Ethics statement

The Institutional Review Board (IRB) at Oklahoma State University approved the survey instruments, and the IRB-approved participant information sheet was used in the consent process. The decisions of the respondents to participate were considered as consent.

### Framework description

The SWOT framework is commonly used in the social sciences to analyze the diversity of perceptions and attitudes among members of heterogeneous groups. In this qualitative method of problem identification, stakeholders are asked to rank positive (strengths and opportunities) and negative (weaknesses and threats) aspects of an issue (e.g., a problem, policy proposal, or business strategy) [23, 24]. In SWOT analysis, internal and external variables are used to describe how different members of a heterogeneous community perceive problems and prioritize solutions to a given issue. Strengths and weaknesses are considered the internal factors and are direct outcomes of a program or priority [23]. Likewise, opportunities and threats are categorized as external factors and are indirect implications of a program [25]. Despite the methodological robustness of the SWOT method, it cannot be employed to generate quantifiable matrices that can be used to rank the importance of SWOT factors [26].

In the AHP, SWOT factors are placed into a hierarchical structure and pairwise comparisons are then employed to rank decisions and determine alternatives [26]. The SWOT-AHP has been extensively used in stakeholder perception analysis concerning natural resource management. For example, Shrestha et al. [23] used the SWOT-AHP method to explore the potential for agroforestry adoption in Florida, and Dwivedi and Alavalapati [27] utilized this method to explore stakeholder opinions on forest biomass-based bioenergy development in the southern United States. Likewise, Stainback et al. [25] explored how stakeholders in Rwanda perceive the agroforestry program being implemented for small landowners using SWOT-AHP analysis. KC et al. [22] also employed this method to investigate the perceptions of community users and experts of the CF program in Nepal.

**Fig 2:**
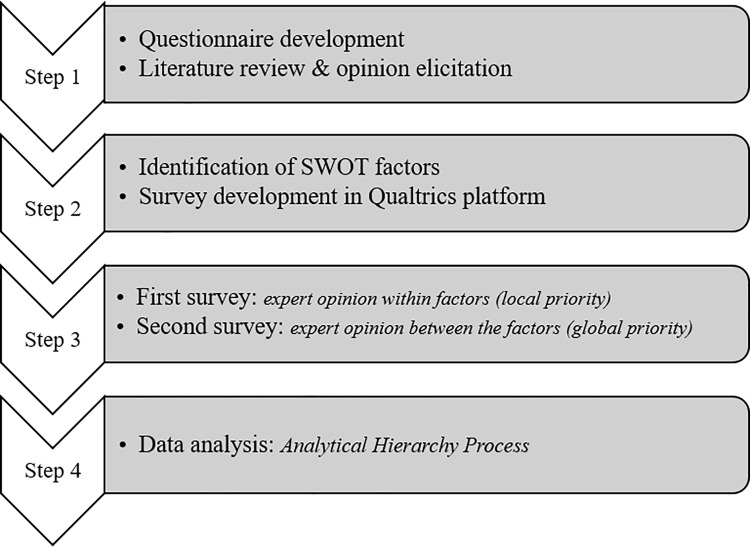
A flowchart of the research process.

In the AHP, pairwise comparisons are performed and matrices are generated that can be used to compare appropriate solutions. The following steps suggested in the SWOT-AHP literature were used for data collection and analysis in this study. A flowchart showing the entire research process is shown in Fig 2.

Step 1: This step involved the questionnaire development. During this process, six knowledgeable experts were requested to provide their feedback on what they thought could be the strengths, weaknesses, opportunities, and threats associated with SFM implementation in Nepal. Their feedback, past experience of the researchers, as well as the available literature, was used to develop appropriate factors under each SWOT category [27]. Categories that were considered helpful for SFM implementation were grouped into strengths and opportunities and the hindrances were categorized into weaknesses and threats.

Step 2: A questionnaire was designed to receive opinion from professionals affiliated with government agencies, NGOs, academic institutions, and forestry advocacy groups. After discussion with knowledgeable persons in Nepal, we selected 30 respondents who had: a) representation to the organization that is involved in forestry sector development; or b) academic background in forestry. The first segment of the questionnaire included questions related to the institutional affiliation of the survey participant and his or her familiarity with and opinion regarding SFM programs. The second segment asked the respondents to rank SWTO attributes related to the SFM implementation in existing CFs and CBFs in Nepal. The relevant factors identified in each SWOT category that reveal stakeholder perceptions of SFM implementation in Nepal are described in Table 1.

**Table 1:** Relevant factors identified in each SWOT category to reveal stakeholder perceptions on SFM implementation in Nepal.

**Strength**	**Weakness**
Financially attractive	Inadequate manpower
Improved stand productivity	Lower community involvement
Reduced fire and other risk and hazard	Corruption
Reduced foreign dependence on wood products	Lack of appropriate technology for harvesting and logging
**Opportunities**	**Threats**
Wood crisis mitigation	Policy and legal uncertainty
Wood-based employment	Low stakeholder support(i.e., FECOFUN)
Rural development	Market uncertainty
Reduced illegal logging	Less supporting infrastructure (road networks, mills)

**Fig 3:**
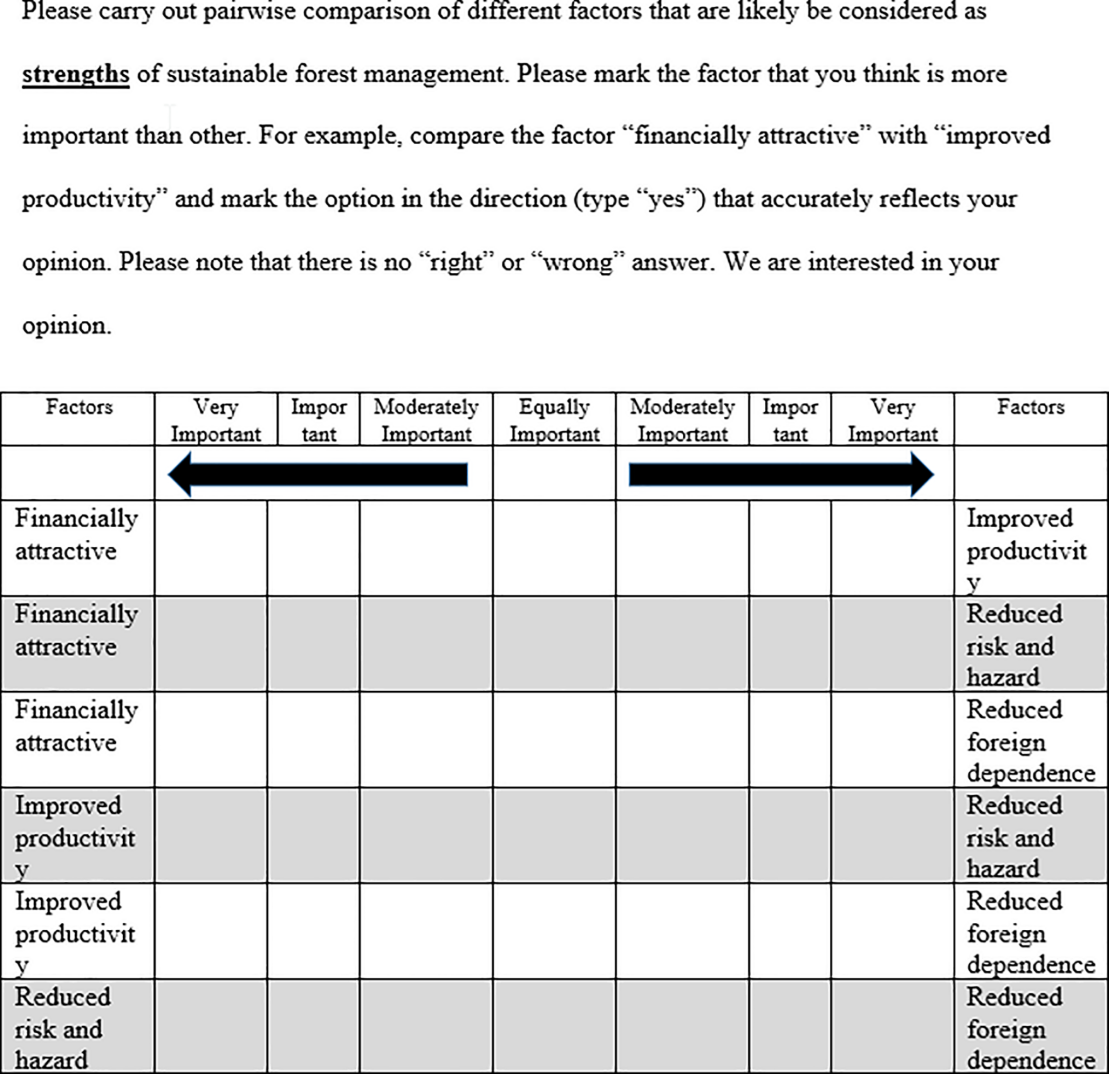
Example of the pairwise comparison of factors within a strength category.

As is typically done in SWOT analysis, the respondents were asked to make pairwise comparisons within the same category in accordance with their perceived importance of factors. For example, within the strengths category, the survey respondents were asked to mark the magnitude of the factor “financially attractive” compared to the factor “improved productivity” using a Likert scale from 1 to 7 (1 = “Equally Important,” 3 = “Moderately Important,” 5 = “Important,” and 7 = “Very Important”). As outlined by Saaty [28], even numbers (2, 4, and 6) serve as intermediate values between adjacent judgments. The same process was used for the weakness, opportunity, and threat categories. An example of a pairwise comparison of factors within the strength category is provided in Fig 3.

Step 3: The questionnaire was designed in Qualtrics and administered as an Internet-based survey by sending an email request to stakeholders that collectively represented the government, NGOs, community forestry advocacy groups, and research institutions. Thirty respondents representing each stakeholder group filled out the survey. Since SWOT-AHP does not require a large sample size for a statistical best fit [27], the opinions of only the 20 experts who were very familiar with SFM implementation were used in the analysis. While the stakeholders representing community forestry advocacy groups revealed their assessment via email, they did not complete the pairwise comparisons. Therefore, only the pairwise comparisons made by the 20 professionals working in the government, research community, and NGOs could be further analyzed using the AHP procedure. It is important to note that consistency ratio (CI) of the results, not the sample size, is the criteria to ensure reliability of the AHP based study finding (e.g. Shrestha et al. 2004). As highlighted by Margles et al. (2010), consistency ratio within 10% is generally considered acceptable and ratio that goes more than 20% requires re-examination of the judgement.

The pairwise comparisons of the stakeholders between factors were determined using the eigenvalue method, as suggested by Saaty [28]. In the pairwise comparisons of a reciprocal matrix, the matrix element *a*_ij_ and weights of relative priorities *w* were expressed as

$$A=a_{ij}=\left(\begin{array}{ccc} \frac{w_{1}}{w_{1}} & \frac{w_{1}}{w_{2}}\cdots & \frac{w_{1}}{w_{n}}\\ \vdots & \vdots & \vdots \\ \frac{w_{n}}{w_{1}} & \cdots & \frac{w_{n}}{w_{n}} \end{array}\right)$$ (1)

The multiplication of matrix A times weight W resulted in the following relationship:

AW=nW (2)

where *n* is the number of rows and W represents the weight of each row (*w*_1_, *w*_2_ ,..*w*_*n*_). Eq 2 can be further expressed as

W(A−nI)=0 (3)

In Eq 3, I is an identity matrix of size *n*. Saaty [28] proved that the principal eigenvector *λ*_max_ of a positive pairwise comparison matrix is equal to *n*. This condition is necessary and sufficient for pairwise comparison, and non-adherence to this assumption may lead to inconsistencies. Therefore, the matrix needs to be checked for consistency as follows:

$$CI=\frac{\left(\lambda_{max}-n\right)}{n-1}$$ (4)

A CI value of less than 10% is assumed to provide a consistent matrix. A clear understanding of the issue among stakeholders, good survey design, objectively revealed opinions, and a relatively small number of factors within the group can help improve (lower) the CI [27, 28].

Step 4: After obtaining information within each category in the pairwise comparisons, the data were analyzed and the factor with the highest priority score was identified in each category. Given the complexity of the topic, the stakeholder groups were employed to differentiate the highest priority scores. Stakeholder group-specific questionnaires were developed and administered among 15 professionals representing all of the stakeholder categories. Eight responses (3 NGO, 3 Government, 2 researchers) were obtained and analyzed following step 3, and global priority scores were obtained accordingly.

## Results and discussion

Table 2 presents the SWOT factors with the respective overall priority values for all three stakeholder groups. Among the stakeholder groups, the government professionals and NGO activists have the desired consistency (CI ≤ 10%) for all tradeoffs within each SWOT category. The researchers also have the desired level of consistency for all tradeoffs except the opportunity category, which has a slightly poorer consistency then desired (CI = 16%). As in previous studies [29, 30], disagreement among stakeholders can lead to higher inconsistency.

**Table 2:** Priority scores of all SWOT factors and categories.

SWOT categories	Factor priority			Overall priority		
	Research professionals	NGO	Government	Research professionals	NGO	Government
**Strengths**				***0*.*398***	***0*.*231***	***0*.*417***
**S1**	0.345	**0.358**	0.182	0.137	0.083	0.076
**S2**	**0.420**	0.326	**0.434**	0.167	0.075	0.181
**S3**	0.121	0.157	0.151	0.048	0.036	0.063
**S4**	0.114	0.159	0.234	0.045	0.037	0.098
**Weaknesses**				***0*.*091***	***0*.*182***	***0*.*206***
**W1**	0.188	**0.382**	0.284	0.017	0.069	0.058
**W2**	0.187	0.103	0.146	0.017	0.019	0.030
**W3**	**0.400**	0.263	0.131	0.036	0.048	0.027
**W4**	0.225	0.253	**0.439**	0.021	0.046	0.090
**Opportunities**				***0*.*086***	***0*.*344***	***0*.*214***
**O1**	0.218	0.113	0.161	0.019	0.039	0.034
**O2**	**0.314**	**0.439**	0.235	0.027	0.151	0.050
**O3**	0.226	0.255	0.139	0.020	0.088	0.030
**O4**	0.242	0.192	**0.466**	0.021	0.066	0.100
**Threats**				***0*.*425***	***0*.*243***	***0*.*163***
**T1**	**0.491**	**0.480**	0.172	0.209	0.117	0.028
**T2**	0.180	0.243	0.289	0.076	0.059	0.047
**T3**	0.163	0.111	0.145	0.069	0.027	0.024
**T4**	0.165	0.166	**0.394**	0.070	0.040	0.064

S1: financially attractive; S2: improved productivity; S3: reduced risk and hazard; S4: reduced foreign dependence; W1: inadequate manpower; W2: lower community development; W3: corruption; W4: lack of harvesting technology; O1: wood crisis mitigation; O2: wood-based employment; O3: rural development; O4: reduced illegal logging; T1: policy/legal uncertainty; T2: low stakeholder support; T3: market uncertainty; T4: less supporting infrastructure.

In Table 2, the local and global priority scores associated with each stakeholder group are reported. The local priority scores, which sum to one, were derived from the direct ratings of the individual factors within each SWOT group. The global priority scores were obtained from the procedures outlined in step 4. The consistency ratio for comparison between the SWOT categories is well below the desired level of 10%.

The local priority of each individual factor within one category was multiplied with the share of the global priority score of that category. The priority scores of the strengths and opportunities were combined and interpreted to have positive values, and the sums of the weakness and threat scores were interpreted to have negative priority values [22, 27, 31]. The itemized perceptions of each stakeholder group regarding SFM implementation in Nepal are presented in detail below.

### Within factor priorities

There were some differences among stakeholders concerning their priorities for strengths, weaknesses, opportunities and threats. For example, among the researchers and government professionals, improved forest productivity received the highest priority as a strength of SFM implementation. The NGO professionals, in contrast, felt that financial attractiveness was the most important strength. Reduced foreign dependence was the second-highest priority among the government professionals, whereas the other stakeholders did not prioritize it as the most important strength of SFM. Notably, Nepal currently imports more than 80% of its timber due, in large part, to existing harvesting restrictions that undermine the need for silvicultural operations in productive tropical timberlands [19]. Furthermore, some stakeholders, as outlined in the existing literature [2, 9, 19] believe that stringent forestry regulations and restrictive community forestry inventory guidelines are the major impediments to overcoming the underutilization of forest resources in Nepal. Such contrasting opinions are evident in the results of this study as well.

All stakeholders had varied opinion concerning weaknesses of SFM implementation. The experts representing government agencies believed that lack of harvesting technology would be the primary weakness. Inadequate manpower and the corruption were cited as the primary weaknesses of SFM implementation by NGO and research professionals. Interestingly, the government professionals provided a substantially lower rating for corruption as a weakness category. In contrast, weakness was rated the highest among the categories for this group. Sal (*Shorea robusta*) is a dominant timber species and the most valuable species in most of the Terai forests. A single tree may be worth several hundred dollars [32]. In addition, institutional corruption in the forestry sector has become an important topic of research and discussion among independent researchers [2, 33]. Since increased timber harvesting, a primary goal of SFM implementation, will result in greater timber revenue, concerns about institutional corruption in non-government groups reflect the prevalent skepticism of government agencies in the country.

Among the opportunities, wood-based employment received the highest priority among the researchers and NGO professionals and was ranked the second-highest by the government professionals. SFM is expected to generate employment opportunities during harvesting, transportation, log conversion, and fuelwood generation [14]. Given that SFM, as is true with any form of active forest management [9], is more likely to result in additional employment opportunities, a broader consensus on employment opportunities across stakeholder groups was not surprising. Moreover, the government stakeholder group identified reduced illegal logging as the most important opportunity factor. Illegal logging is a serious issue in productive sal forests in the Terai region [2, 33]. Government professionals, with the help of special armed force guards, are responsible for controlling illegal logging and confiscating illegally obtained timber [2, 14, 33]. With the implementation of SFM, the movement and monitoring of forestry workers, including forest management crew and professionals, may eventually increase, which might facilitate the control of illegal activities in the SFM implementation area.

Similar to opportunities, experts representing NGOs and research community had similar opinion such that both stakeholders believed policy and legal uncertainty being the most important threat. The government professionals, in contrast, thought that less supporting infrastructure could be the most challenging threat for long-run implementation of SFM policies. The researchers and NGO professionals were most concerned about the existing policies and legal uncertainty of SFM. Because Nepal is currently undergoing political transformation, policies and legal uncertainties are credible threats to the implementation of any program in Nepal. For example, despite formal endorsement of the SFM guidelines in 2014 [14], their implementation was briefly halted by the parliamentary Committee on Environment [21].

### Between factor priorities

**Fig 4:**
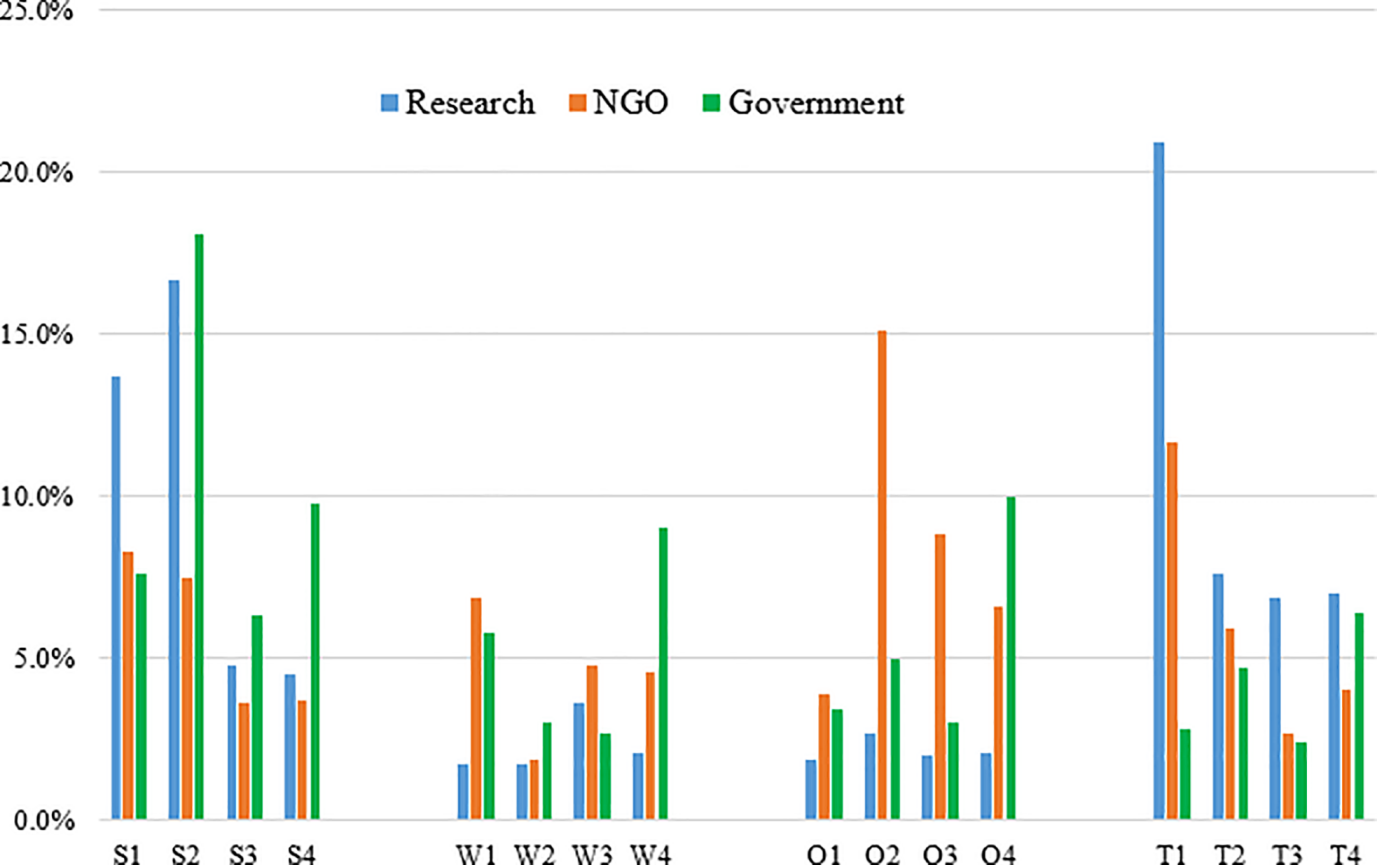
A bar diagram depicting differences between stakeholder groups in terms of their overall priorities given to the factors. S1: financially attractive; S2: improved productivity; S3: reduced risk and hazard; S4: reduced foreign dependence; W1: inadequate manpower; W2: lower community development; W3: corruption; W4: lack of harvesting technology; O1: wood crisis mitigation; O2: wood-based employment; O3: rural development; O4: reduced illegal logging; T1: policy/legal uncertainty; T2: low stakeholder support; T3: market uncertainty; T4: less supporting infrastructure.

The government group perceived improved productivity, lack of harvesting technology, reduced illegal logging, and less supporting infrastructure as the most important strength, weakness, opportunity, and threat, respectively. The combined positive priority value of this stakeholder group, which was the sum of scores for strength and opportunity factors [27], was found to be 63% (Table 2). As shown in Table 2, the strength category (42%) dominated the overall perception of the government stakeholder group, followed by the opportunity (21%) and weakness (21%) categories. The threat category received the lowest score. The government professionals ranked improved productivity (18%) as the main strength of SFM in Nepal (Fig 4). Under the weakness category, the two highest priorities were given to lack of harvesting technology (9%) and inadequate professional manpower (6%). Among the opportunity factors, reduced illegal logging (10%) received the highest score. Moreover, the government stakeholder group perceived less supporting infrastructure (6%) and low stakeholder support (5%) as the two major threats to SFM in Nepal. An intuitive explanation behind this observation may be that government foresters are being pragmatic in responding to this question as they may have already experienced logistic challenges regarding technology and manpower, but are knowledgeable enough to appreciate the production potential of timberland under new management scenario. While CF has been globally admired for its innovation as a co-management practice [2], it has been criticized for being a passive form of forest management that promotes protection-oriented management [9]. Since these stakeholders represented foresters having formal training in forestry, their higher acceptance towards SFM, a form of active forest management that may lead to timber productivity [9], is not surprising.

Likewise, the combined positive value for the NGO group was found to be 58%. The opportunity category (34%) was the most important determinant of the overall perception of the NGO professionals (Fig 4). The second most important category was threat (24%), followed by strength (23%). The NGO group gave the lowest score to the weakness category. Among the strength factors, financially attractive (8%) and improved productivity (8%) received the highest scores. Inadequate professional manpower (7%) and corruption (5%) were the two most important weakness factors identified by this group. Similarly, among the opportunity factors, wood-based employment (15%) was given the highest score, followed by rural development (9%). The NGO stakeholders also identified policy/legal uncertainty (12%) as a main threat to SFM implementation in Nepal. While many NGOs in Nepal are involved in natural resource advocacy and public mobilization movements, they also play an important role in facilitating the service delivery of government forestry programs [8]. As such, their positive opinion concerning SFM implementation would help ensure transfer of knowledge to the grassroots.

Our survey results show that the research professionals perceived improved productivity, corruption, wood-based employment, and policy/legal uncertainty as the most important strength, weakness, opportunity, and threat, respectively (Table 2). The overall priority values yield a combined positive value of 48%, indicating that the overall perception of the research professionals was slightly negative regarding SFM implementation in Nepal. While researchers have generally applauded the motivation behind SFM implementation, some scholars within the social science realm are skeptic of and view that it might result in poor forest governance due in part to corruption and managerial inefficiencies [21].

The overall perception of the research professionals was mostly determined by threats (42%) followed by strengths (40%). The research professionals ranked policy/legal uncertainty (21%) as the top threat (Fig 4). Given the changing political climate of the country and inherent policy hurdles in SFM implementation[14], concerns expressed by research community are reasonable and need to be addressed to ensure its effectiveness [21]. Improved productivity (17%) and financial attractiveness (14%) were the two major strengths that research professionals perceived regarding SFM implementation in Nepal. Since extensive silvicultural operations and active management will result into higher timber removal from SFM implemented forests [34], these results make intuitive sense. The weakness category explained slightly over 9% of the overall perception of this group. Among the weakness factors, the research professionals provided the highest score to corruption (4%) followed by lack of harvesting technology (2%). Furthermore, the research professionals ranked the opportunity category the lowest, as it explained approximately 9% of the overall perception. Nonetheless, in the opportunity category, the highest score was given to wood-based employment (3%) followed by reduced illegal logging (2%).

Overall, study results suggest that all professional stakeholders perceived that the strengths of the existing SFM policies are more important than their inherent weaknesses. There was across-the-board agreement regarding the need for better management of forest resources in Nepal [9]. Therefore, our study results corroborate the narrative that protection-oriented management needs to be changed to meet the increased timber demand of the country.

A few limitations of this work are worth noting. While this study was focused on the perceptions of SFM implementation by stakeholders, it did not focus on their insights into the causes of the existing passive forest management in Nepal. SFM implementation cannot be successful without diagnosing the root causes of ineffectiveness within existing management regimes. Likewise, despite our deliberate efforts to engage forest user groups in the survey conducted in this study, only a handful of incomplete responses were available; therefore, they could not be included in the AHP model. One participant expressed concern that SFM could undervalue the principles of sustainable forest management in Nepal. Such concerns suggest that there is inherent skepticism among forest user groups concerning SFM policy. Nonetheless, because user groups receive training and other capacity-building support from NGOs and academic groups, the collective opinions of the non-state stakeholders were adequately incorporated in our analysis.

### Management and policy implications

The study results provide important insights into SFM implementation in Nepal. First, meaningful community participation is important for sustainable forest resource management. As our results reveal, NGO and research professionals differ from other stakeholders in their opinions regarding the positive and negative aspects of SFM implementation. SFM implementation will not be successful if stakeholder opinions are not incorporated into policy formulation. Likewise, government forestry professionals should assist community forestry users by developing guidelines that are easy to comprehend and implement.

Second, some stakeholders, particularly the activists representing community forestry user groups, have expressed concerns over growing corruption in timber sales [21]. Although corruption is a legitimate concern in Terai forest management [13], it is an issue of governance and can be minimized through government transparency and accountability [35]. Our study results revealed that other stakeholders are not assured of good governance and accountability issues within the existing SFM mechanisms. SFM implementation will only succeed if other stakeholders, including those representing CFUGs, are able to internalize the need for scientific intervention in the existing forest management programs and work together to deliver good governance.

Third, while this analysis was conducted using data from stakeholders in the Nepalese forestry sector, the study results have broader policy implications as the decentralization of forest governance has resulted in new forest use practices in many countries around the world [36]. Other Asian countries share similar political and institutional arrangements with Nepal and can take lessons from this study regarding the socio-political dynamics associated with innovative management strategies like SFM. Given the exemplary past success of decentralized forest governance [1–3], the initiatives of Nepal regarding SFM implementation contain important lessons for other countries.

## Conclusion

Despite an abundance of forest resources, passive forest management in Nepal has led to significant timber importation from other counties. Unfortunately, passive forest management activities have led Nepal to import more than 80% of the timber necessary to meet its domestic demands [19]. Therefore, SFM implementation is a step toward enabling Nepal to self-sustain its timber demands. These efforts are timely, as the nation requires more timber now to meet the housing demands that have exponentially increased in the aftermath of the 2015 earthquake [20]. The study results suggest that there is an across-the-board consensus regarding the need for active forest management in Nepal. However, there are subtle differences among stakeholder concerns regarding the SWOT of SFM implementation. For example, while government professionals considered a lack of supporting infrastructure to be the most important SFM implementation bottleneck, the other stakeholders were more concerned about corruption. In short, this study provides empirical evidence that government professionals need to work together with other stakeholders to institutionalize SFM implementation. While the stakeholder perception analysis was focused on SFM implementation in Nepal, these study results could have implications for other countries that have practiced the participatory model of forest governance.

## Supporting information

S1 File The IRB approved survey instrument used to collect first round of data. (DOCX)

S2 File The IRB approved survey instrument used to collect second round of data. (DOCX)

S3 File The data used to generate relevant tables and figures. (XLSX)
