# Supplementary material for: Stakeholder opinions on scientific forest management policy implementation in Nepal
Source: PLoS One. 2018 Sep 5;13(9):e0203106. doi: 10.1371/journal.pone.0203106 (PMC6124746; doi:10.1371/journal.pone.0203106)
Supplement: S1 File — (DOCX) [file pone.0203106.s001.docx]

**Stakeholder Opinion on Scientific Forest Management Implementation in Nepal**

**Section A**: In this section, we would like to know your familiarity with scientific forest management

A1. Which of the following types of organizations do you belong to? (**CHECK (√) FOR ALL THAT APPLY**)

□ **Department of Forest**

□ **Government organization other than department of forest**

□ **FECOFUN**

□ **Community forestry user group**

□ **Non-government organization**

□ **International non-government organization**

□ **University**

□ **Other (please specify ____________________)**

A3. How familiar are you with the concept of Scientific Forest Management (SFM)? (Please **CHECK ONE**)

□ **Very familiar** □ **Somewhat familiar**

□ **Uncertain** □  **Not familiar**

A4. What is your opinion about SFM Implementation in Nepal? **(CHECK (√) FOR ALL THAT APPLY**)

□ Important for sustainable wood supply

□ Important for economic prosperity

□ Both

□ None

□ Not sure

**Section B**: In this section, we ask you to rank strength, weakness, opportunities, and threats related to scientific forest management implementation in Nepal. Please utilize table below to compare different attributes associated with sustainable forest management.

| **Strength** | **Weakness** |
| --- | --- |
| Financially attractive | Inadequate manpower |
| Improved stand productivity | Lower community involvement |
| Reduce fire and other risk and hazard | Corruption |
| Reduced foreign dependence on wood products | Lack of appropriate technology for harvesting and logging |
| **Opportunities** | **Threats** |
| Wood crisis mitigation | Policy and legal uncertainty |
| Wood based employment | Low stakeholder support(i.e. FECOFUN) |
| Rural development | Market uncertainty |
| Reduced illegal logging | Less supporting infrastructure (road network, mills) |

1. **Strength**

Please carryout pairwise comparison of different factors that are likely be considered as **strength** of sustainable forest management. Please mark the factor that you think is more important than other. For example, compare the factor “financially attractive” with “improved productivity” and mark the option in the direction (type yes) that accurately reflects your opinion. Please note that there is no ‘right’ or ‘wrong’ answer. We are interested to seek your opinion.

| Factors | Very  Important | Important | Moderately  Important | Equal  Important | Moderately  Important | Important | Very  Important | Factors |
| --- | --- | --- | --- | --- | --- | --- | --- | --- |
|  |  | | |  |  | | |  |
| Financially attractive |  |  |  |  |  |  |  | Improved productivity |
| Financially attractive |  |  |  |  |  |  |  | Reduced risk and hazard |
| Financially attractive |  |  |  |  |  |  |  | Reduced foreign dependence |
| Improved productivity |  |  |  |  |  |  |  | Reduced risk and hazard |
| Improved productivity |  |  |  |  |  |  |  | Reduced foreign dependence |
| Reduced risk and hazard |  |  |  |  |  |  |  | Reduced foreign dependence |

1. **Weakness**

Please carryout pairwise comparison of different factors that are likely be considered as **weakness** of sustainable forest management. Please mark the factor that you think is more important than other. For example, compare the factor “inadequate manpower” with “lower community involvement” and mark the option in the direction (type yes) that accurately reflects your opinion. Please note that there is no ‘right’ or ‘wrong’ answer. We are interested to seek your opinion.

| Factors | Very  Important | Important | Moderately  Important | Equal  Important | Moderately  Important | Important | Very  Important | Factors |
| --- | --- | --- | --- | --- | --- | --- | --- | --- |
|  |  | | |  |  | | |  |
| Inadequate manpower |  |  |  |  |  |  |  | Lower community involvement |
| Inadequate manpower |  |  |  |  |  |  |  | Corruption |
| Inadequate manpower |  |  |  |  |  |  |  | Lack of harvesting technology |
| Lower community involvement |  |  |  |  |  |  |  | Corruption |
| Lower community involvement |  |  |  |  |  |  |  | Lack of harvesting technology |
| Corruption |  |  |  |  |  |  |  | Lack of harvesting technology |

1. **Opportunities**

Please carryout pairwise comparison of different factors that are likely be considered as **opportunities** of sustainable forest management. Please mark the factor that you think is more important than other. For example, compare the factor “wood crisis mitigation” with “wood based employment” and mark the option in the direction (type yes) that accurately reflects your opinion. Please note that there is no ‘right’ or ‘wrong’ answer. We are interested to seek your opinion.

| Factors | Very  Important | Important | Moderately  Important | Equal  Important | Moderately  Important | Important | Very  Important | Factors |
| --- | --- | --- | --- | --- | --- | --- | --- | --- |
|  |  | | |  |  | | |  |
|  |  |  |  |  |  |  |  |  |
| Wood crisis mitigation |  |  |  |  |  |  |  | Wood based employment |
| Wood crisis mitigation |  |  |  |  |  |  |  | Rural development |
| Wood crisis mitigation |  |  |  |  |  |  |  | Reduced illegal logging |
| Wood based employment |  |  |  |  |  |  |  | Rural development |
| Wood based employment |  |  |  |  |  |  |  | Reduced illegal logging |
| Rural development |  |  |  |  |  |  |  | Reduced illegal logging |

1. **Threats**

Please carryout pairwise comparison of different factors that are likely be considered as **threats** of sustainable forest management. Please mark the factor that you think is more important than other. For example, compare the factor “policy/legal uncertainty” with “low stakeholder support” and mark the option in the direction (type yes) that accurately reflects your opinion. Please note that there is no ‘right’ or ‘wrong’ answer. We are interested to seek your opinion.

| Factors | Very  Important | Important | Moderately  Important | Equal  Important | Moderately  Important | Important | Very  Important | Factors |
| --- | --- | --- | --- | --- | --- | --- | --- | --- |
|  |  | | |  |  | | |  |
| Policy/legal uncertainty |  |  |  |  |  |  |  | Low stakeholder support |
| Policy/legal uncertainty |  |  |  |  |  |  |  | Market uncertainty |
| Policy/legal uncertainty |  |  |  |  |  |  |  | Less supporting infrastructure |
| Low stakeholder support |  |  |  |  |  |  |  | Market uncertainty |
| Low stakeholder support |  |  |  |  |  |  |  | Less supporting infrastructure |
| Market uncertainty |  |  |  |  |  |  |  | Less supporting infrastructure |

Thank you for taking time to fill out our questionnaire. Your cooperation is greatly appreciated. Would you like to be sent a copy of the results of this survey?

□ **Yes □ No.**

Please feel free to write any comments you have in the space below.
