# Supplementary material for: Stakeholder opinions on scientific forest management policy implementation in Nepal
Source: PLoS One. 2018 Sep 5;13(9):e0203106. doi: 10.1371/journal.pone.0203106 (PMC6124746; doi:10.1371/journal.pone.0203106)
Supplement: S2 File — (DOCX) [file pone.0203106.s002.docx]

Questionnaire 2-Expert opinions on scientific forest management implementation in Nepal.

Few weeks ago, stakeholders representing government, non-government and community forestry user groups participated in a survey that aimed to understand strengths, weaknesses, opportunities, and threats related to scientific forest management being implemented in some Terai Districts of Nepal. The highest ranked strengths, weaknesses, opportunities, and threats are:

Strength [S1]: xxxxxxx

Weakness [W1]:xxxxxx

Opportunity [O1]:xxxxxx

Threat [T1]:xxxxxx

Now I ask you to compare the highest ranked factors in each category. For example, compare the Strength factor [S1] with the weakness factor [W1] and mark the option in the direction that accurately reflects your opinion. Please note that there is no ‘right’ or ‘wrong’ answer. I am interested to seek your opinion.

| Factors | Very  Important | Important | Moderately  Important | Equal  Important | Moderately  Important | Important | Very  Important | Factors |
| --- | --- | --- | --- | --- | --- | --- | --- | --- |
|  |  | | |  |  | | |  |
| S1 |  |  |  |  |  |  |  | W1 |
| S1 |  |  |  |  |  |  |  | O1 |
| S1 |  |  |  |  |  |  |  | T1 |
| W1 |  |  |  |  |  |  |  | O1 |
| W1 |  |  |  |  |  |  |  | T1 |
| O1 |  |  |  |  |  |  |  | T1 |

Interested readers can reproduce all three surveys using the information available from Table 2.
